# Bevacizumab treatment for neovascular age-related macular degeneration in the setting of a clinic: “real life” long-term outcome

**DOI:** 10.1186/s12886-015-0019-x

**Published:** 2015-04-11

**Authors:** Gala Beykin, Michelle Grunin, Edward Averbukh, Eyal Banin, Yitzchak Hemo, Itay Chowers

**Affiliations:** Department of Ophthalmology, Hadassah - Hebrew University Medical Center, PO Box 12000, Jerusalem, 91120 Israel

**Keywords:** Neovascular age-related macular degeneration, Anti-vascular endothelial growth factor, Bevacizumab, Long-term

## Abstract

**Background:**

To evaluate the long-term outcome of bevacizumab therapy for neovascular age related macular degeneration (NVAMD) in the setting of a clinic.

**Methods:**

Consecutive group of NVAMD patients who were treated in a single 3^rd^ referral center with bevacizumab using a loading dosage of 3 monthly injections followed by variable dosing for at least 48 months were retrospectively evaluated. Genotyping was performed for CFH (rs1061170), HTRA1 (rs1200638), and C3 (rs2230199). Main outcome measures included functional and morphological treatment outcomes as well as their risk allele associations.

**Results:**

Out of 128 patients who started bevacizumab treatment over 4 years before the study endpoint [mean (±SD): 60 ± 10.9 months], 75 eyes of 67 (52.3%) patients, were still followed. Mean best corrected visual acuity (BCVA) (LogMAR ± SEM) improved from 0.66 ± 0.07 at baseline to 0.48 ± 0.05 (p = 0.012) at 1 year, but deteriorated from the 3^rd^ year on and at the final exam reduced to 0.69 ± 0.07 (p = 0.6, compared with initial BCVA). Macular thickness mirrored visual acuity (VA) changes showing initial thinning followed by thickening from the 3^rd^ year on. Individuals carrying the CFH risk -allele had a mean thickening (microns ± SEM) of 66.9 ± 70.4 versus a mean thinning of 76.8 ± 22 in non-carriers (p = 0.015).

**Conclusions:**

Bevacizumab therapy for NVAMD using a flexible treatment algorithm in a “real life” clinical setting initially obtained VA gain and thinning of the macula that were maintained for two years, but were lost later on.

## Background

Multiple clinical trials have documented improved short- and intermediate term visual acuity (VA) following application of anti-vascular endothelial growth factor (VEGF) therapy in neovascular age related macular degeneration (NVAMD) [1-4]. Such visual gain was obtained for up to two years using bevacizumab, ranibizumab or aflibercept utilizing either fixed or pro re nata (PRN) regimens of intraocular injections [1,4,5].

Limited data is available on the long-term outcome of VEGF blockade in NVAMD, and this data focuses on ranibizumab [6-11]. It has been suggested that most of the visual gain obtained during the first one to two years of treatment may be lost later on. The Open-Label Extension Trial of Ranibizumab for Choroidal Neovascularization Secondary to Age-Related Macular Degeneration (HORIZON) provides follow-up for two additional years of PRN ranibizumab treatment among patients who previously had completed two years within the ANCHOR, MARINA, or FOCUS trials. The study reported on 361 patients and showed that at four years following initiation of therapy the majority of visual gain obtained in the first months of therapy was lost [9]. The Seven-Year Outcomes in Ranibizumab-Treated Patients in ANCHOR, MARINA, and HORIZON (SEVEN-UP) study recently provided the longest currently available follow-up of seven years after initiation of ranibizumab treatment. Sixty-five patients out of the 357 patients originally randomized to ranibizumab in the ANCHOR or MARINA trials who also completed two years of participation in the HORIZON study were re-examined. A mean loss of 8 letters compared with the initial VA was reported, and vision loss appears to progress with time for some of the eyes. Forty-six percent of the eyes that were re-examined were still receiving anti-VEGF treatment [6].

The long-term safety of ranibizumab 0.5 mg in neovascular age-related macular degeneration (SECURE) study, which provided an additional 24 months of follow up after completion of a 12-month treatment period of two other (EXCITE and SUSTAIN) studies, also showed that a visual acuity-guided regimen of ranibizumab administration led to a best corrected visual acuity (BCVA) reduction from study baseline [8].

In the clinic setting, Pushpoth et al. reported that 50% of 110 NVAMD patients who were treated with ranibizumab for NVAMD and followed for four years maintained visual acuity (VA) of at least 0.3 [11]. Rasmussen and colleagues reported of stable VA of approximately 0.3 decimal in 192 NVAMD eyes treated with ranibizumab for 4 years [10].

Advances in genetics over the last few years inspired the idea that genetic testing-guided personalized medicine may become a reality in ophthalmology. Single nucleotide polymorphisms (SNPs) and rare variants in over 20 genes are currently known to be associated with the risk for manifesting AMD [12-16]. Yet, conflicting information was reported with respect to the existence of pharmacogenetic interactions among these variants in the context of anti-VEGF therapy [17-21], and long-term data on this issue is lacking.

We aimed to assess the long-term outcome of bevacizumab therapy for NVAMD in the “real life” setting of a clinic (rather than a controlled study) and to evaluate for potential pharmacogenetic interactions. To that end, NVAMD patients who underwent continuous treatment for at least 4 years were evaluated for final VA, anatomical response, and potential interaction of outcomes with genotyping for the major risk SNPs associated with AMD.

## Methods

A single center retrospective study was performed. All NVAMD patients who presented between January 2006 to January 2008 to the retina clinic of the Hadassah Medical Center and treated for at least 48 months with either intravitreal bevacizumab alone or in combination with ranibizumab injections were identified by computer search of the clinic database. Institutional review board approval was obtained.

Data recorded included demographics (age and gender), ophthalmic findings including BCVA on an Early Treatment Diabetic Retinopathy Study (ETDRS) chart, and choroidal neovascularization (CNV) characteristics according to fluorescein angiogram (FA). Morphological characteristics of the lesion before and during follow-up were assessed using optical coherence tomography (OCT; stratus OCT [Carl Zeiss Meditec, Inc. Dublin, CA] for all case up to 2009 and Spectralis HRA-OCT [Heidelberg Engineering, Heidelberg, Germany]) from 2009 and on. Imaging scans were assessed and analyzes by a masked observer. A Correction factor of 70 microns was added to the Stratus OCT measurements to allow calculation along with the Spectralis OCT driven values [22].

The treatment algorithm included three consecutive monthly intravitreal injections of 1.25 mg bevacizumab, followed by an OCT-guided individualized treatment regimen for at least 48 months. Clinical examination and OCT were performed following each injection. In some cases, when treatment failure under bevacizumab was detected, second line therapy with ranibizumab was attempted. Need for retreatment and drug switch was determined by the treating physician (IC, EA, EB, IH) based on assessment of disease activity according to VA, ophthalmoscopy, and OCT, as well as FA when required. OCT guidelines used for retreatment included persistence or recurrence of either intra-retinal, sub-retinal or sub-retinal pigment epithelium fluid. These guidelines were similar for all four physicians. New subretinal hemorrhage was also mandated re-treatment. In case where the visual acuity dropped but the OCT failed to demonstrate fluid, fluorescein angiography was performed and re-treatment was provided if active CNV was demonstrated. While no specific lower visual acuity threshold for treatment was determined, treatment was withheld in eyes with visual acuity lower than 20/200 where the treating physician felt that additional therapy would be futile due to irreversible macular damage. Guidelines for switching treatment from bevacizumab to ranibizumab included persistence or recurrence of either intra-retinal, sub-retinal or sub-retinal pigment epithelium fluid despite at least three monthly bevacizumab injections. These were based upon the clinical judgment of the treating retina specialists for each individual patient.

Outcome was evaluated by changes in ETDRS VA and OCT findings from baseline. OCT parameters evaluated included central subfield thickness (CST) and central point thickness (CPT), as well as the presence of intra-retinal, sub-retinal, sub-retinal pigment epithelium fluid or atrophy. Atrophy was determined by macular thinning and by absence of the photoreceptor inner/outer segment (IS/OS) junction in the central macular area. FAs were evaluated to determine the type and location of the lesion. Lesions were measured using the OIS WinStation XP 5000™ (MediVision Medical Imaging, Yokneam Elit, Israel). Genotyping was performed in 45 patients of the 67 patients included in the study. These patients signed an IRB-approved informed consent form for this purpose. The three major risk SNPs for AMD including rs1061170 in complement Factor H (CFH), rs1200638 in high temperature requirement A1 (HTRA1) and rs2230199 in complement component 3 (C3) were evaluated via sequencing as previously described [13,14,23].

Statistical analysis was performed using the SPSS Statistics software, version 22.0 (IBM Corporation, Somers, NY). The Paired Student *t* test was utilized to analyze quantitative variables such as the VA and macular thickness across different time points compared with the initial findings. Prevalence of categorical parameters was compared using Fisher’s exact test, and correlations were assessed using the Pearson test. Statistical significance was defined as a P value of less than 0.05.

Approval for this work by the Institutional Review Board (Helsinki Committee) of the Hadassah Medical Center was obtained. The study was performed with informed consent.

## Results

Between January 2006 and January 2008, intravitreal bevacizumab treatment for NVAMD was initiated in 142 eyes of 128 patients at the retina clinic of Hadassah medical center. In 67 eyes of 61 patients, treatment was shorter than 4 years due to death (n = 16), loss to follow-up (n = 31) or clinical judgment of stable condition or an untreatable state (n = 14). These 14 patients were not included as they were not considered candidates for anti-VEGF treatment since they manifested lesions predominated by either subfoveal scaring or atrophy associated with poor visual acuity. Baseline demographics and ocular characteristics for 75 eyes of 67 patients treated for at least 48 months and recruited for this study are shown in Table 1.

**Table 1:** **Baseline patient demographical and clinical characteristics**

**Patient characteristics**	**No. (%)**	**Mean ± SEM**
Gender		
Female	41 (61.2)	
Male	26 (38.8)	
Age (y)		77.48 ± 0.91
Age group		
<70 y	12 (17.9)	
70-80 y	27 (40.3)	
>80 y	28 (41.8)	
Using AREDS supplements	41 (61.2)	
Smoking		
Yes	7 (10.4)	
No	37 (55.2)	
Past	12 (17.9)	
Missing	11 (16.4)	
Hypertension		
Yes	47 (70.1)	
No	19 (28.4)	
Missing	1 (1.5)	
Diabetes mellitus		
Yes	17 (25.4)	
No	48 (71.6)	
Missing	2 (3)	
Ischemic heart disease		
Yes	18 (26.9)	
No	47 (70.1)	
Missing	2 (3)	

SEM = standard error of the mean; y = years.

Initial characteristics and final outcomes are summarized in Table 2. Twenty two of the eyes were previously treated with photodynamic therapy (PDT) and were switched to bevacizumab therapy once available; the remaining 53 eyes were treatment naïve. Mean total lesion size (largest linear dimension ± SEM) measured 3184 ± 231.48 microns in the 50 eyes (66.7%) in which this parameter was available. CNV was classified to either classic (including predominantly classic lesions) or occult (including minimally classic lesions) in 16 (27.1%) and 43 (72.9%) eyes, respectively. Mean lesion size (largest linear dimension ± SEM) measured 2330 ± 278 microns and 3417 ± 291 microns in classic and occult lesions, respectively. None of the patients had polypoidal choroidal vasculopathy or retinal angiomatous proliferation. Mean follow-up time (±SD) was 60 ± 10.9 months (range: 48–90 months), during which the average number of anti-VEGF injections (±SD) was 27 ± 12.7 per eye or 5.4 injections per eye per year. The 25^th^, 50^th^ and 75^th^ percentiles of cumulative injections per eye were 16, 26 (median) and 36, respectively.

**Table 2:** **Baseline and final study eye characteristics**

	**Baseline**		**Final**		
	**No. (%)**	**Mean ± SEM**	**No. (%)**	**Mean ± SEM**	**P value**
LogMAR BCVA		0.66 ± 0.07		0.69 ± 0.07	0.600
BCVA (approximate Snellen equivalent)		20/90		20/100	
20/200 or worse	13 (17.3)		15 (20)		
Better than 20/200 but worse than 20/40	34 (45.3)		33 (44)		
20/40 or better	28 (37.3)		27 (36)		
Initial BCVA (ETDRS letters)					
<45	66 (88)		66 (88)		
≥45	9 (12)		9 (12)		
CPT (microns)		369.8 ± 12.5		335.9 ± 25.3	0.146
CST (microns)		378.4 ± 10.1		355.4 ± 20.2	0.142
Intra-retinal fluid	47 (65.3)		23 (32)		0.038
Sub-retinal fluid	55 (76.4)		21 (29.2)		0.125
Pigment epithelial detachment	25 (34.7)		16 (22.2)		0.770
Scar	13 (18.1)		37 (51.4)		0.013

BCVA = best corrected visual acuity; CPT = central point thickness; CST = central subfield thickness; ETDRS = Early Treatment Diabetic Retinopathy Study; error of the mean; SEM = standard error of the mean. Baseline OCT parameters were available for 72 eyes.

**Figure 1:**
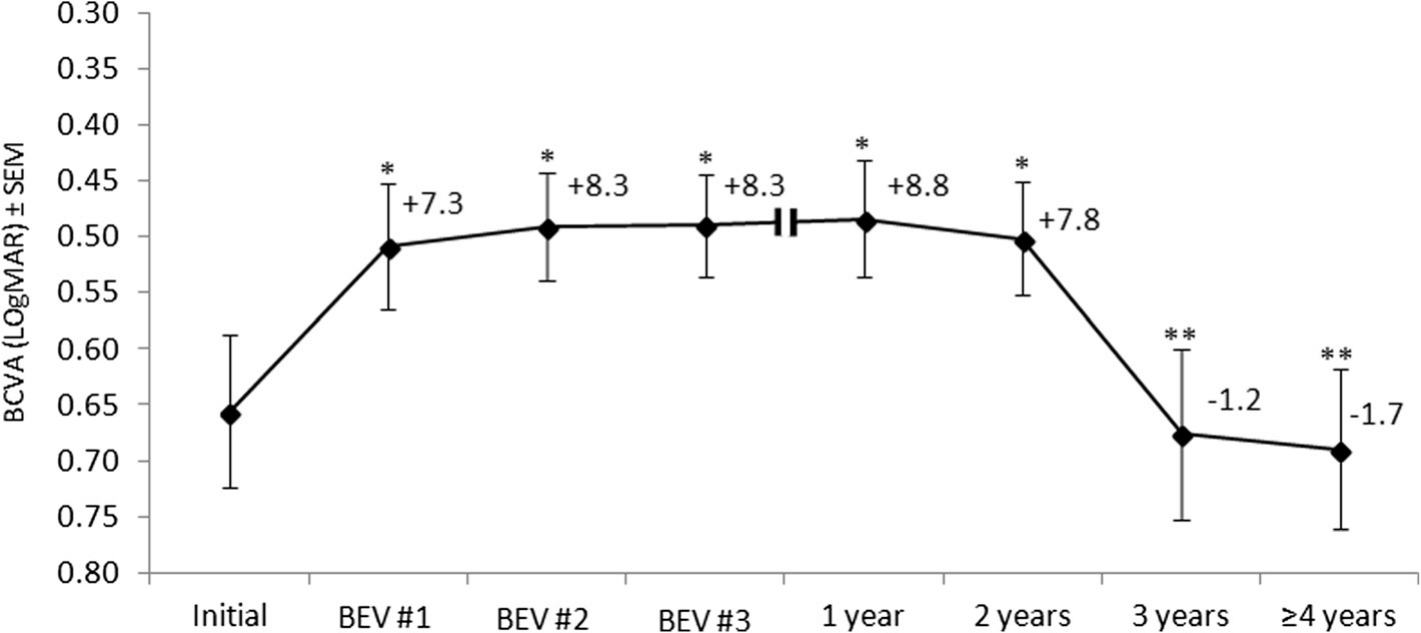
The mean change in LogMAR visual acuity score (±SEM) during follow-up (n = 75). Mean number of ETDRS letters gained or lost versus baseline visual acuity is shown at each time point. BCVA = best corrected visual acuity; BEV #1, #2, #3 = first, second and third bevacizumab injections, respectively; ETDRS = Early Treatment Diabetic Retinopathy Study; SEM = standard error of the mean. *p < 0.05 vs. initial; **p < 0.05 vs 2 years.

Baseline mean LogMAR BCVA (±SEM) was 0.66 ± 0.07 (equivalent to approximately 20/90). Mean change in LogMAR at each time-point compared with initial BCVA is shown in Figure 1. VA improved from baseline to a mean of 0.5 ± 0.05 (equivalent to approximately 20/60 or an improvement of 7.8 ETDRS letters; p = 0.044) at 24 months. From this point on, VA reduced to 0.68 ± 0.08 LogMAR (p = 0.026; compared with VA at 24 months) and 0.69 ± 0.07 LogMAR (approximately 20/95; p = 0.03; compared with VA at 24 months) at 36 months and at last follow-up, respectively. There was no significant difference between the initial and final VA obtained after a minimum of 4 years. Subgroup analysis of 34 eyes with an initial LogMAR BCVA of 0.4 or less (equivalent to 20/50 or better) demonstrated a reduction of VA from a mean initial (LogMAR ± SEM) of 0.23 ± 0.02 to 0.40 ± 0.05 at the end of follow-up (p = 0.0001).

Patients previously treated with PDT had an initial BCVA (LogMAR ± SEM) of 0.83 ± 0.16 versus 0.58 ± 0.07 in treatment naïve patients (p = 0.1). Final VA in eyes previously treated with PDT (n = 22 eyes) was 0.86 ± 0.17 compared with 0.62 ± 0.07 in treatment naive eyes (n = 53 eyes; p = 0.15).

Twenty four eyes were switched to ranibizumab when treatment failure under bevacizumab was detected by the treating physician. These eyes received a mean (±SEM) of 28.9 ± 2.1 (range: 7–54) bevacizumab injections and a mean (±SEM) of 7.5 ± 2.03 ranibizumab injections. VA and thickness parameters outcome (CPT and CST) did not differ between bevacizumab only (51 eyes) and ranibizumab switched (24 eyes) eyes (data not shown).

**Figure 2:**
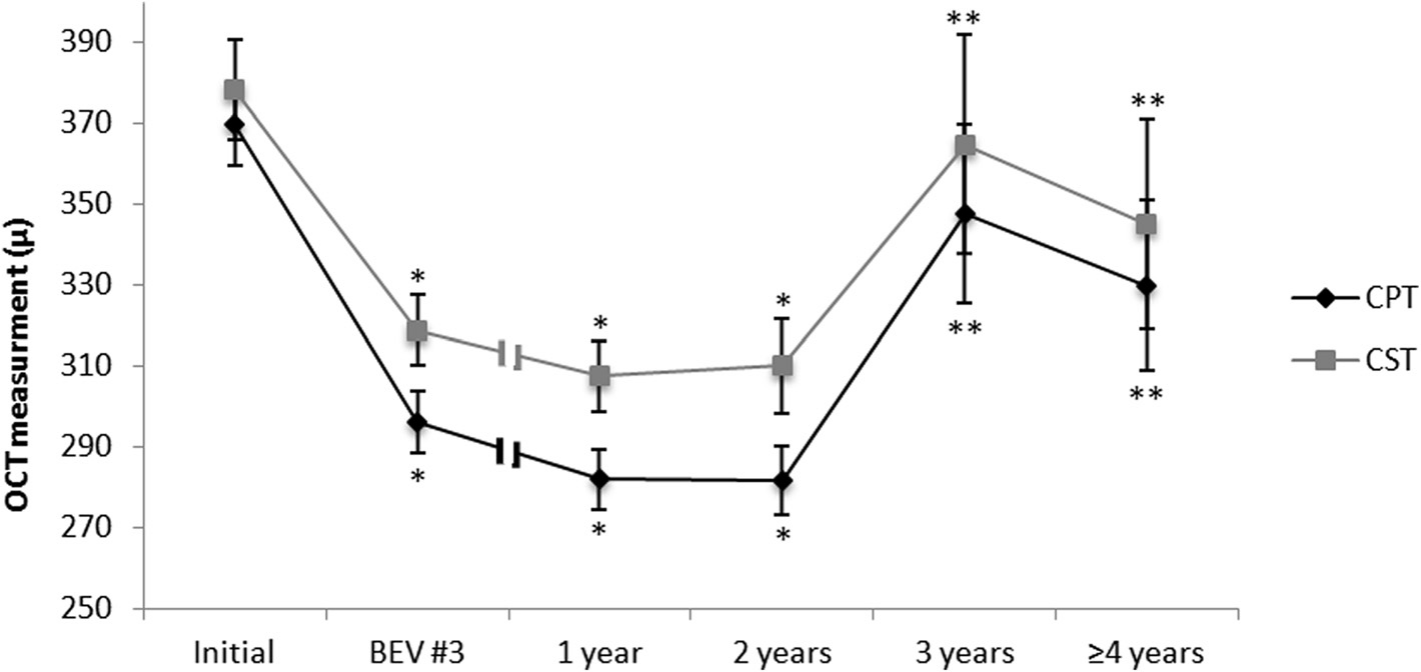
The mean change (±SEM) in central point thickness (CPT) and central subfield thickness (CST) measurements along the follow up period. BEV #3 = third bevacizumab injection; OCT = optical coherence tomography; *p < 0.05 vs. initial; **p < 0.05 vs. 2 years.

Mean CPT and CST prior to treatment and during therapy are shown in Figure 2. Mean baseline CPT and CST (microns ± SEM) were 369.8 ± 12.5 and 378.4 ± 10.1, respectively. Mean CPT and CST reduced significantly after 3 injections and remained lower than baseline after two years of therapy. Thereafter, the thickness increased and was similar to baseline levels at 36 months and at last follow up, reaching a final CPT value of 335.9 ± 25.3 and CST of 355.1 ± 20.2, (p = 0.15 and p = 0.14 vs. initial thickness, respectively).

Evaluation for a possible association between VA and retinal thickness parameters (CPT, CST) was performed in individuals with an abnormally thickened macula. Thinned or thickened macula was defined as CST and CPT of less or over two SD than normal average value, respectively [24]. A direct correlation was found between the change in BCVA and the change in CPT in a subgroup of 34 eyes with final CPT of ≥ 275 μ (Pearson rho = 0.41, p = 0.017). Similar findings were demonstrated in a subgroup of 33 eyes with final CST ≥ 320 μ (Pearson rho = 0.42, p = 0.014). We found that 25.3% of the eyes showed CPT of less than 180 μ, which is subnormal for the OCT system utilized [24]. VA was not associated with such abnormally thinned macula.

Analysis of the OCT images at baseline and at the end of follow-up revealed that prevalence of intra-retinal fluid decreased by approximately 50% and prevalence of sub-retinal fluid showed a trend for decreasing at final exam compared with baseline. On the other hand, scar tissue was almost 3-times more common at the end of follow-up compared with baseline. Pigment epithelial detachment showed similar prevalence at baseline and at last follow up (Table 2). Eyes with atrophy at last exam (n = 39) (absence of IS/OS and thinned macula) showed a trend towards having a lower final VA compared with eyes with no atrophy identified (n = 36; 0.80 ± 0.1 vs 0.56 ± 0.09 LogMAR, p = 0.09). Similar proportion of eyes with and without atrophy at last exam were previously treated with PDT.

No other correlations were detected between VA or thickness parameters and age, gender, number of injections, lesion characteristics according to FA, presence of atrophy or fluid according to OCT, or any of the other parameter which were evaluated.

The risk alleles for AMD in CFH (rs1061170), HTRA1 (rs1200638) and C3 (rs2230199) SNPs were not associated with mean VA change or initial or final VA. However, the CFH variant was associated with mean change in macular thickness parameters. Initial CPT and CST (microns ± SEM) were 375.5 ± 19.2 and 387.4 ± 16.1 in individuals lacking this risk allele (n = 31) and 379.4 ± 30.1 and 393.5 ± 25.9 in risk allele carriers (n = 13), respectively (p = 0.91 and p = 0.84, respectively). At the end of follow up, the CPT and CST decreased by a mean (microns ± SEM) of 92.6 ± 26.4 and 76.8 ± 22.0 respectively, in individuals lacking this risk allele. By contrast, in carriers of the risk allele CPT and CST increased by a mean of 79.9 ± 74.9 (p = 0.009, vs non-carriers) and 66.9 ± 70.4 (p = 0.015, vs non-carriers). Final CPT was 459.3 ± 76.8 in risk allele carriers and 284.4 ± 24.0 in non-carries (p = 0.007) and final CST was 460.3 ± 67.5 and 312.0 ± 17.4 (p = 0.006), respectively (Table 3). The CFH risk allele carriers and non-carriers did not differ in terms of previous PDT treatment (23.1% versus 25.8% received PDT treatment, respectively; p = 1.0), total number of anti-VEGF injection (22.31 ± 4.29 versus 27.06 ± 2.03, respectively; p = 0.3), ranibizumab treatment (38.46% versus 29.03%, respectively; p = 0.7) or number of ranibizumab injections (1.62 ± 0.81 versus 1.97 ± 0.80, respectively; p = 0.7).

**Table 3:** **Frequency of alleles and genotypes of CFH Rs1061170, HTRA1 Rs11200638 and C3 Rs2230199 SNPs among patients**

	No. (%)*	**Final CPT (μ) ± SEM**	**P value**	**Final CST (μ) ± SEM**	**P value**	**Change in CPT (μ) ± SEM**	**P value**	**Change in CSF (μ) ± SEM**	**P value**
**CFH**									
**TT (WT)**	**31 (70.5)**	**284.4 ± 24.0**	**0.007**	**312.0 ± 17.4**	**0.006**	**−92.6 ± 26.43**	**0.009**	**−76.8 ± 22.01**	**0.015**
**CC + TC (AT RISK)**	**13 (29.5)**	**459.3 ± 76.8**		**460.3 ± 67.5**		**79.9 ± 74.84**		**66.9 ± 70.38**	
**HTRA1**									
**GG (WT)**	**14 (35.9)**	**378.1 ± 35.7**	**0.57**	**378.1 ± 27.0**	**0.79**	**2.57 ± 35.83**	**0.49**	**−8.29 ± 32.75**	**0.669**
**AA + GA (AT RISK)**	**25 (64.1)**	**338.4 ± 47.8**		**362.8 ± 40.7**		**−47.54 ± 50.50**		**−36.21 ± 45.38**	
**C3**									
**CC (WT)**	**27 (60)**	**331.1 ± 40.6**	**0.95**	**364.2** **± 36.4**	**0.87**	**−52.04 ± 42.88**	**0.814**	**−29.81 ± 40.89**	**0.964**
**GG + GC (AT RISK)**	**18 (40)**	**326.6 ± 63.2**		**354.1 ± 50.7**		**−33.63 ± 70.62**		**−26.6 ± 61.255**	

BCVA = best corrected visual acuity; CPT = central point thickness; CST = central subfield thickness. SEM = standard error of the mean; WT = wild type. *Genotyping results were available for 44, 39 and 45 patients (out of 45 patients tested) for CFH, HTRA1 and C3, respectively.

## Discussion

In this study we analyzed the long-term outcome of intravitreal bevacizumab therapy for NVAMD in a “real-life” clinical setting. Approximately half of the eyes that started therapy more than 4 years prior to the study endpoint were still followed in our clinic and were included in the analysis. Number of patients included and drop-out rate from the study favorably compare with the few reports available in the literature on this topic, such as the SEVEN-UP study [6]. Mean visual acuity improved from one through 24 months following initiation of treatment in a magnitude similar to that obtained in the pivotal clinical trials ANCHOR and MARINA [2,3]. Yet, this visual gain was lost by the end of a mean follow-up of 5 years. Subgroup analysis demonstrated substantial final visual loss in individuals who had initial VA of 20/50 or better. Macular thickness parameters demonstrated a similar course showing initial thinning of the macula, followed by re-thickening after the two year time point as well as an increase in the degree of atrophic changes.

Limited data exist on the long-term outcome of anti-VEGF therapy in NVAMD, particularly in a “real-life” (versus study-controlled) setting, and previous reports on this topic focused on ranibizumab-treated patients [6,7,9-11]. In four years of follow-up in the ANCHOR and MARINA extension study, the HORIZON, there was an incremental decline of the VA gains seen during the first 2 years of the studies. The recent SEVEN-UP trial reported on re-examination of approximately 10% of the original ANCHOR and MARINA cohorts. Since enrollment to ANCHOR and MARINA trials, approximately 7 years previously, a mean loss of 8.6 letters was reported for these patients [6]. Among the factors that may underlie such visual loss are under- or over-treatment, decaying medication effect (i.e. tolerance or tachyphylaxis), and the development of macular scarring and atrophy.

The treatment algorithm in our study, as well as in the other long-term reports [6-11], was clinician determined. Unlike patients included in the SEVEN-UP [6] study that were originally treated in the ANCHOR and MARINA trials, our patients were not treated initially by a fixed monthly regimen. Furthermore, some of our patients were previously treated with PDT and some were switched to second-line ranibizumab therapy. The SECURE study [6] suggested that under-treatment may be associated with poorer visual outcome at 3 years in patients treated based on VA [8]. In the SEVEN-UP study, better visual outcomes were demonstrated among patients in the highest quartile of anti-VEGF treatments. That study suggested that the low treatment frequency of 1.6 injections per year since exit from HORIZON study, may have contributed to the decline in mean VA [6]. However, in the HORIZON trial, the mean number of ranibizumab injections received by patients who had stable or improved VA at last observation (a mean of 4.2 injections) was lower than the number received by patients with worse final VA outcome (a mean of 4.9 injections) [9].

In the clinic setting, Pushpoth et al. reported an average of 5.8 and 13.7 ranibizumab injections by 12 and 48 months, respectively (approximately 3.4 injections per year). Fifty percent of the patients maintained Snellen VA of at least 0.3 decimal over 4 years in this study [11]. Rasmussen et al., utilizing a strategy of three initial monthly ranibizumab injections and an overall mean of 5.5 ranibizumab injections per year during four years of follow-up, reported of stable baseline and final VA (approximately 0.3 decimal) [10]. In our study, a similar clinician-determined treatment algorithm was applied for bevacizumab, resulting in a similar frequency of 5.4 injections per year.

Our results demonstrate similar deterioration from the 3^rd^ year of treatment as seen in the HORIZON, an open-label, multicenter study and the SEVEN-UP, a multicenter, nonintervention cohort study, and show comparable initial and final VA as reported from the clinic setting studies [6,9-11]. These data suggest that in the long-term, bevacizumab therapy results in outcomes similar to those obtained with ranibizumab therapy. Furthermore, our data suggest that the deterioration observed after 2 years of therapy in the ANCHOR and MARINA trials [2,3] cannot be entirely attributed to a switch from a fixed monthly to a clinician determined treatment strategy.

Tolerance or tachyphylaxis to anti-VEGF compounds may also have a role in this poor long-term effect. Long-term loss or reduced efficacy was reported for non-ocular biological therapy [25-27]. Such phenomenon was also described for anti-VEGF therapy in NVAMD [28], and its prevalence in 14 to 24 months of follow-up was reported to be between 2% to 10% [29,30]. The contribution of tachyphylaxis and tolerance to the long-term loss of the initial visual gain in treated NVAMD patients is still unknown. Macular thickening accompanying visual loss from the 3^rd^ year of therapy and on was demonstrated in our study. Yet, thickening was accompanied by decreased presence of intra-retinal fluid and increased prevalence of macular scarring. These data suggests that insufficient VEGF blockade due to insufficient frequency of injections or loss of their efficacy may play a role in long term VA loss, but, that other causes of fibrosis may also at least partially account for such visual loss.

Another potential major cause of visual loss in the long-term is the development of atrophic macular changes. Such atrophy may be caused by the primary progression of the degeneration associated with AMD, or secondary to the presence and regression of the CNV. It is also possible that anti-VEGF therapy in itself results in accelerated atrophy, as VEGF may have a role as a neuronal survival factor and its long-term suppression could affect retinal health. The SEVEN-UP study reported that 57 of the 58 eyes (98%) examined by fundus autofluorescence imaging had macular atrophy. The same study demonstrated that decreased VA is associated with both increased area of macular atrophy and presence of subfoveal macular atrophy [6]. The CATT study reported of a higher rate of atrophy development in the monthly anti-VEGF treated groups than in the PRN treated groups [1]. We have estimated atrophy based on thinning of the CPT and by absence of the photoreceptors-IS/OS junction in the central macular area as demonstrated on OCT imaging. We found that macular thinning or attenuation of the IS/OS line showed borderline association with worse long-term VA outcome.

A correlation between the presence of the major AMD risk allele in CFH and a worse outcome in terms of OCT thickness parameters is another finding of the present study. The association of this SNP with worse outcome was inconsistently reported in other relatively short term studies which evaluated this issue [18,31]. For example, the CATT trial and the IVAN trial concluded that the major risk alleles for AMD did not predict response to anti-VEGF therapy by one year of follow-up [18,31]. It is noteworthy that our study describes a long-term association of genotyping and outcome while IVAN and CATT trials [18,31] describe a shorter time outcome. In the long-term, outcome may reflect atrophic changes or other factors that are less relevant in the short term. In addition, a recent review by Kanoff et al. [19], concluded based on previous reports [32-35] that presence of the high-risk C allele in the CFH Y402H locus seems to increase both a patient’s risk of macular degeneration and of a worse treatment response to anti-VEGF therapy [19]. Further research is required to explore the significance of such associations.

Several limitations of this study should be acknowledged. This study includes a relatively small number of eyes and it is retrospective, and almost 50% of cases in which treatment was initiated were not available for the long-term (>4 years) follow up. Some of the eyes were previously treated with PDT and some received second-line ranibizumab therapy. However, this cohort contains a group of patients treated for a mean of 5 years by the same physicians in the same facility. Similar previous studies [6,9-11] reported follow-up rate ranges from about 55% in the HORIZON study to as low as 10% in both the SEVEN-UP study and the study by Pushpoth and colleagues [11], underscoring the difficulty in obtaining higher rates of follow up for such a long-term in this elderly and highly variable patient population.

## Conclusions

Our results show that three sequential monthly injections of bevacizumab followed by a clinician-determined treatment strategy led to marked VA gain which was maintained for two years, and then subsequently gradually lost. Sub-optimal treatment strategy, development of atrophy and scarring, and diminishing treatment efficacy (or some combination of these factors) may explain this phenomenon. Larger prospective studies assessing long-term outcome are required to provide insight to the mechanisms resulting in visual deterioration in NVAMD despite a good initial response to anti-VEGF therapy.
